# Brillouin microscopy monitors rapid responses in subcellular compartments

**DOI:** 10.1186/s43074-024-00123-w

**Published:** 2024-04-10

**Authors:** Zachary N. Coker, Maria Troyanova-Wood, Zachary A. Steelman, Bennett L. Ibey, Joel N. Bixler, Marlan O. Scully, Vladislav V. Yakovlev

**Affiliations:** 1 Department of Physics & Astronomy, Texas A&M University, 4242 TAMU, College Station, TX 77843 USA; 2 SAIC, Fort Sam Houston, TX 78234 USA; 3 Air Force Research Laboratory, JBSA Fort Sam Houston, Fort Sam Houston, TX 78234 USA; 4 Institute for Quantum Science and Engineering, Texas A&M University, College Station, TX 77843 USA; 5 Department of Biomedical Engineering, Texas A&M University, 3120 TAMU, 101 Bizzell Street, College Station, TX 77843 USA

**Keywords:** Microscopy, Imaging, Brillouin scattering, Raman scattering, Fluorescence

## Abstract

Measurements and imaging of the mechanical response of biological cells are critical for understanding the mechanisms of many diseases, and for fundamental studies of energy, signal and force transduction. The recent emergence of Brillouin microscopy as a powerful non-contact, label-free way to non-invasively and non-destructively assess local viscoelastic properties provides an opportunity to expand the scope of biomechanical research to the sub-cellular level. Brillouin spectroscopy has recently been validated through static measurements of cell viscoelastic properties, however, fast (sub-second) measurements of sub-cellular cytomechanical changes have yet to be reported. In this report, we utilize a custom multimodal spectroscopy system to monitor for the very first time the rapid viscoelastic response of cells and subcellular structures to a short-duration electrical impulse. The cytomechanical response of three subcellular structures - cytoplasm, nucleoplasm, and nucleoli - were monitored, showing distinct mechanical changes despite an identical stimulus. Through this pioneering transformative study, we demonstrate the capability of Brillouin spectroscopy to measure rapid, real-time biomechanical changes within distinct subcellular compartments. Our results support the promising future of Brillouin spectroscopy within the broad scope of cellular biomechanics.

## Introduction

The mechanical properties of living cells have become increasingly recognized as key to regulating biological function, with deep implications for such diverse concerns as cancer development [1–5], viral infections [6, 7], chronic illnesses like asthma [8], and even aging [9, 10]. Examples of the importance of biomechanical cues are legion - glaucoma, for example, is associated with changes in the optic nerve extracellular matrix which drives fibrosis and stiffening [10] and is linked with increased intraocular pressure driven by changes in endothelial cell viscoelasticity [11]. Cancer cells have been observed to be more compliant both before and during tumorigenesis [1, 2] and loss of plasticity and reduced stiffness have been identified as cellular biomarkers for both the presence and metastatic potential of thyroid, ovarian, and breast cancer [12]. Recent studies also point to a new paradigm in cancer therapeutics, focusing on targeted modifications of cytoskeletal structure or membrane rigidity, which combats the invasive potential of cancer cells [13–15].Numerous other examples may be found in extensive review articles which highlight the important connections between cellular biomechanics and cellular function [16–20].

The importance of cellular biomechanics has spurred the creation of numerous experimental approaches which attempt to measure key mechanical properties of cells. Atomic force microscopy (AFM) [21], profile microindentation [22], magnetic twisting cytometry [23], particle tracking microrheology [24], and optical stretching [25] are only some of the methods used to investigate cell and tissue biomechanics. A challenge of nearly all modern techniques for investigating biomechanics is that they are highly invasive methods which typically alter or destroy the sample during measurement, either through physical deformation or the introduction of exogenous agents. This invasive perturbation of the sample may be circumvented by optical rheology techniques. Optical coherence elastography (OCE), for example, has become prevalent as a non-destructive tool for tissue-level studies, however, OCE still requires mechanical excitations and has limited spatial resolution with conventional systems limited to between 5 and 15 μm [26, 27]. There is a clear need for a rapid, real-time, non-destructive tool for visualizing micromechanical dynamics in living cells [28–30].

A plethora of new developments in the design of Brillouin spectrometers have allowed for a Brillouin renaissance in the last decade, establishing Brillouin spectroscopy as a powerful tool for biomechanical sensing and imaging applications [28, 29, 31]. In this work we have adopted recent advances in spectrometer design to combine Brillouin and Raman spectroscopy, two all-optical non-invasive and label-free techniques, for rapid and continuous monitoring of subcellular mechanical and chemical information. This multi-modal spectroscopy approach enabled us to precisely measure the mechanical dynamics of individual sub-cellular components, and to quantify subtle mechanical changes which are not observable using traditional microscopy with sub-second temporal resolution and sub-cellular compartment specificity. In particular, dynamic changes in the nucleus, nucleolus, and cytoplasm were monitored to visualize changes in response to a short (< 1 µs) high energy electric impulse, which is known to rapidly induce cytoskeletal changes in living cells [32, 33]. This study demonstrates the utility of Brillouin microscopy for rapid, compartment-specific imaging of cellular mechanical changes.

## Principle

Brillouin spectroscopy is an optical technique that relies on inelastic light scattering from acoustic phonons in a medium. In performing such spectroscopic measurements, a monochromatic laser beam is directed towards a sample, and the resulting scattered light is collected and analyzed. By analyzing the frequency shift, called the Brillouin frequency shift (${\nu }_{B}$), and the linewidth of the spectral peak in the scattered light, it is possible to assess the mechanical properties of the sample with high spatial resolution. The main advantages of Brillouin spectroscopy are that it is non-invasive and label-free while allowing for 3D microscopic imaging. This makes it a valuable tool for studying the mechanical properties of biological objects in vivo with diffraction-limited spatial resolution. The Brillouin shift, ${\nu }_{B}$, is related to the acoustic wave velocity ($V$) by ${\nu }_{B}=\pm 2\frac{nV}{\lambda }\text{sin}\left(\frac{\theta }{2}\right)$, where $n$ is the refractive index of the sample, *λ* is the incident laser radiation wavelength, and *θ* is the angle between incident and scattering directions. The largest frequency shift occurs in a backscattered geometry at $\theta =\pi$. The real (elastic) part of the complex high-frequency longitudinal modulus, $M^{\prime }$, is defined by ${M}^{{\prime }}={\left(\frac{{\nu }_{B}\lambda }{2n}\right)}^{2}\rho$, where $\rho$ is a local mass density of the material. The imaginary (viscous) part of the high-frequency longitudinal modulus is defined by ${M}^{{\prime }{\prime }}={\nu }_{B}{{\Gamma }}_{B}{\left(\frac{\lambda }{2n}\right)}^{2}\rho$, where ${{\Gamma }}_{B}$ is the Brillouin line-width. Thus, by measuring the Brillouin frequency shift and the linewidth at the same time with a proper knowledge of the sample (namely, refractive index and mass density), one can deduce both the viscous and elastic modulus of the tissue under study [30]. While recent investigations have demonstrated static tissue, cellular, and sub-cellular mechanical measurements using Brillouin spectroscopy [34–40], its utility for assessing intracellular dynamics in response to external stimuli has not yet been explored, nor have fast dynamic subcellular measurements within the cell nucleus or nucleolus been achieved.

The scientific premise for our study is a previously established change of plasma membrane permeability upon applying a well-defined pulsed electrical stimulus. We hypothesized that mechanical disruptions of sub-cellular structures should follow such an abrupt change and focused on investigating the dynamics of viscoelastic properties inside a cell at different spatial locations associated with different sub-cellular structures. Brillouin microscopy was selected to detect and characterize those changes given its ability to provide viscoelastic contrast, superior spatial resolution, and its non-invasiveness. Upon successful assessment of the fast dynamic changes of local elastic properties, we performed complementary fluorescence microscopy studies to confirm the disruption of cytoskeletal structures and to elucidate the most likely physical cause behind the observed changes of mechanical properties.

## Results

### Static subcellular Brillouin and Raman spectroscopy measurements

We built a custom confocal Raman and Brillouin microscope capable of simultaneously recording chemically- and mechanically specific information from intracellular compartments. Several areas inside CHO-K1 cells were targeted to record Raman and Brillouin spectra and observe changes induced by exposure to a brief, but intense 600 ns electrical stimulus, or nanosecond pulsed electric field (herein referred to as nsPEF), which is known to depolymerize actin and induce biomechanical alterations in living cells [41, 42]. We observed decreased Brillouin frequency shift measurements from within various cellular components following nsPEF exposures. This data is coupled with fluorescence microscopy images showing cell plasma membrane and cytoskeleton disruption that help to explain our results.

**Fig. 1:**
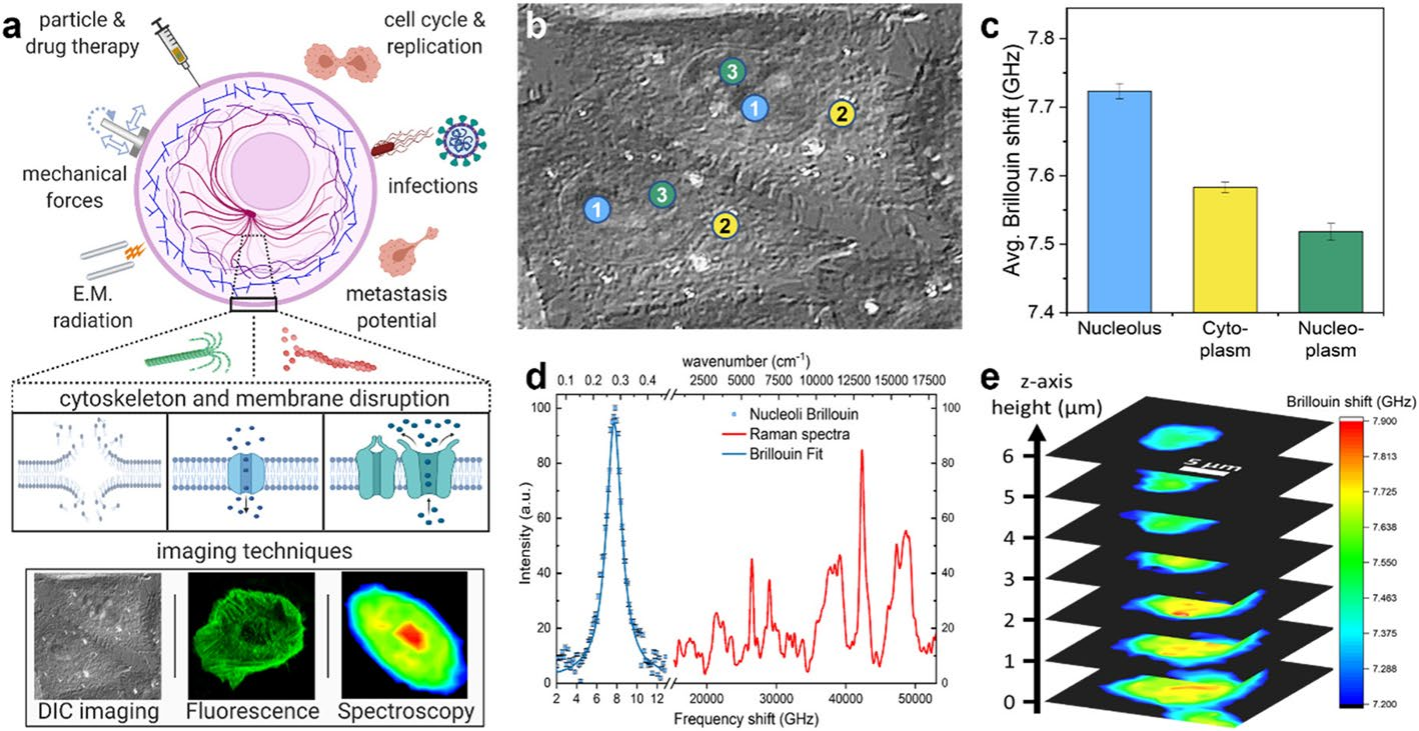
**a** (top) Conceptual illustration with examples of stimuli and cell behaviors linked to changes in cellular mechanics, (middle) cell responses, such as filament depolymerization, membrane disruption, and channel activation, and (bottom) examples of microscopic imaging modalities used in this report. Instrument capabilities and measurements include: **b** DIC imaging, indicators color-matched to **c** providing examples of locations used for Brillouin measurements taken from the subcellular compartments. **c** Average Brillouin frequency shift measurements from each of the three compartments. **d** Representative example of Brillouin and Raman spectral measurements from within a cell nucleolus. **e** Example of 3D mechanical map from CHO-K1 cell via z-stack of 2D Brillouin images. **a** Created with BioRender.com.

The confocal Raman-Brillouin microscopy setup allowed for spectral measurements with submicron scale resolution, providing observations of time-resolved changes at the subcellular level. The subcellular imaging capabilities of the microscope are demonstrated in Fig. 1 along with a conceptual illustration of this experiment. Spectra were collected from three target regions within CHO-K1 cells to verify the system capability for distinguishing between individual subcellular components. The three target regions included the cytoplasm (defined as any location within the cell membrane that was outside of the perinuclear region), the nucleoplasm (inside the nuclear membrane, excluding nucleoli), and the nucleolus. An initial comparison showed that all components within the cell had a greater Brillouin frequency shift as compared to the surrounding media (${\nu }_{media}$ = 7.159 ± 0.021; averaged across multiple measurements per experiment per day). Furthermore, the peak linewidth, measured as full-width half-maximum (FWHM) of a Lorentzian fit exhibits a broadened shape caused by phonon damping that is expected in spectra acquired from heterogeneous materials such as cells [29, 37, 43]. The mean Brillouin shift of each region across all cells was found to be ${\nu }_{B}$ = 7.583 ± 0.007 GHz, 7.518 ± 0.012 GHz, and 7.723 ± 0.011 GHz (*N* = 35, 23, 19) for cell cytoplasm, nucleoplasm, and nucleoli, respectively. The average linewidth across the sample volumes measured ${{\Gamma }}_{B}$ = 1.335 ± 0.017 GHz, 1.233 ± 0.011 GHz, and 1.424 ± 0.017 GHz respectively, indicating that the individual components also have measurably different viscosity. All errors are presented as standard errors of the mean (SEM). The nucleoli were measured to have significantly higher Brillouin frequency shifts (*p* ≤ 0.0001) than the other primarily-protoplasm areas and the cytoplasm was found to have Brillouin frequency shift measurements that were significantly higher than that of the nucleoplasm (*p* < 0.0001).

Raman spectra were acquired during all trials to provide validation of cell targeting and to monitor the focal volume during experiments. A comparison of the first and last Raman spectrum of each sample also provided a way to check for chemical changes within the focal volume as result of the external stimulus. Comparison of these spectra revealed no noticeable differences in Raman peak intensities or peak ratios across all spectra. The average Brillouin frequency shift measurements from each target region are shown Fig. 1c along with an example of typical Brillouin and Raman spectra acquired from cells in Fig. 1d.

Finally, we performed a small section raster scan in the two lateral dimensions to generate a two-dimensional Brillouin image of the Brillouin shift. An example of a 3D composite generated by a z-stack series of such mechanical maps is shown in Fig. 1e. The Brillouin images provide good indication that the individual component measurements are correct, as the 2D maps provide clear visual confirmation of the relative Brillouin shifts and of boundaries within the cell, most notably that of nucleoli and nuclear envelope.

### Dynamic Brillouin microscopy measurements in response to external stimulus

**Fig. 2:**
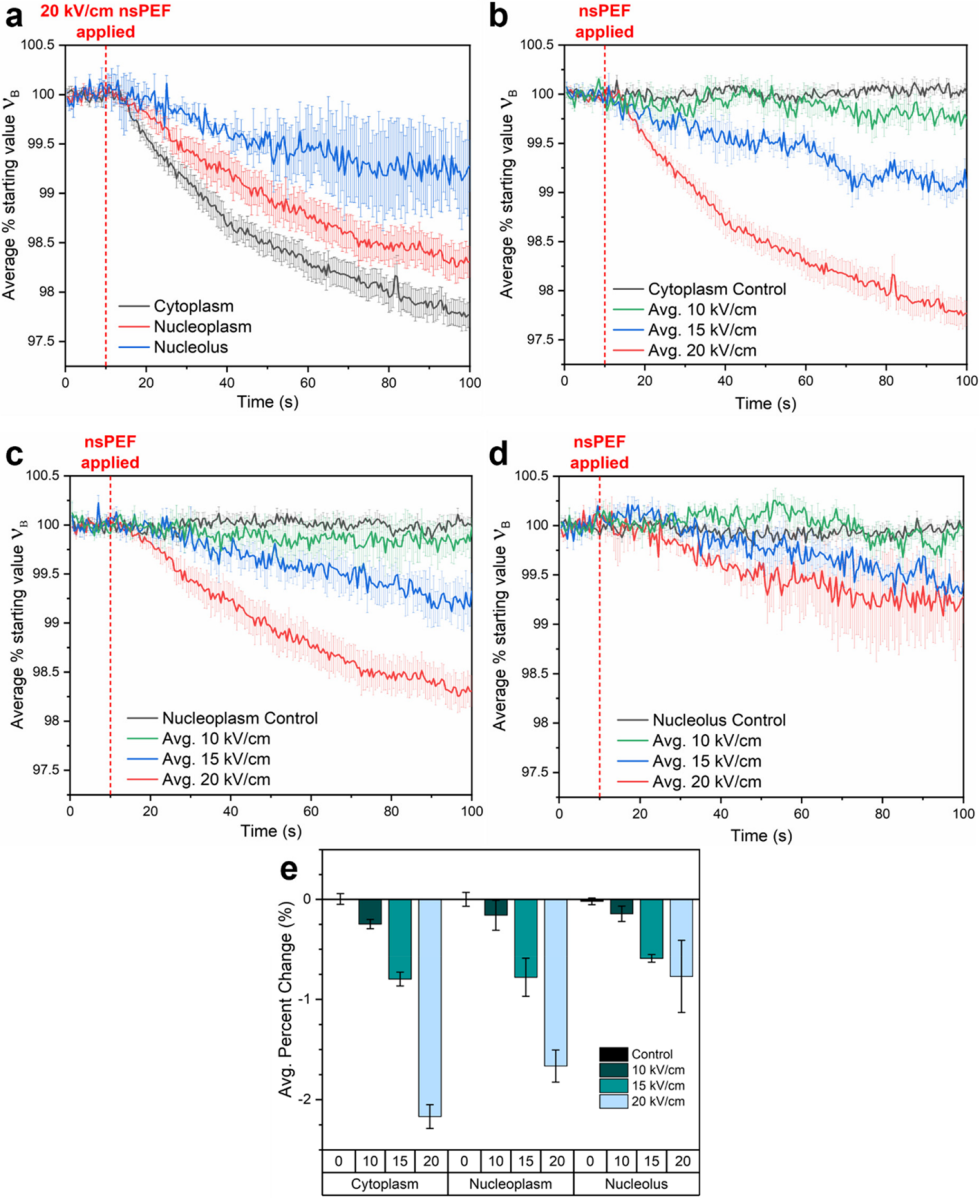
Time-resolved Brillouin frequency shift measurements of (**a**) All three target regions exposed to 20 kV/cm nsPEF, followed by (**b**) cytoplasm (**c**) nucleoplasm (inside the nuclear envelope) and (**d**) nucleolus, with each exposed to 10, 15 and 20 kV/cm. Values are displayed as a percentage of the starting value, as determined by a baseline average of the first 20 points in each series. **e** Bar chart depicting the average percent change by region and field intensity

Brillouin spectral time series from the cytoplasm, nucleoplasm, and nucleoli are shown in Fig. 2a-d. Figure 2a shows the average responses from all three target compartments following the most intense 20 kV/cm nsPEF, while Fig. 2b-d depict the mean percent change in Brillouin shift for the cytoplasm, nucleoplasm, and nucleolus, respectively. For each compartment, the response to 10, 15, and 20 kV/cm nsPEF intensities are reported alongside their respective controls. All cases exhibit a distinct, decreasing, and asymptotic trend following nsPEF stimulus. Changes in the Brillouin frequency shift, and thus longitudinal modulus, become apparent almost immediately following the stimulus (red dashed line). We note that the nucleoli not only presented the greatest control Brillouin shift of the three target regions, but also exhibited the smallest overall change in magnitude after the external stimulus was applied. For example, following the greatest intensity stimulus of 20 kV/cm, we observed an average reduction in Brillouin frequency shift of ${\Delta }{\nu }_{B}$ = 2.168 ± 0.119, 1.664 ± 0.160, and 0.771 ± 0.360% for the cytoplasm, nucleoplasm, and the nucleolus, respectively. A statistically significant difference was found in the final post-exposure Brillouin shift difference measurements (${\Delta }{\nu }_{B}$) for all three compartments against their respective controls (*P* ≤ 0.0001 for both cytoplasm and nucleoplasm and *p* ≤ 0.0005 for nucleoli) for field intensities of 15 and 20 kV/cm. Only the cytoplasm retained a significant difference (*p* ≤ 0.005) at the lowest tested 10 kV/cm field intensity. The reduction in ${\Delta }{\nu }_{B}$ corresponds to an overall increase in cellular mechanical compliance (i.e., a reduction of the longitudinal modulus) [36] and is consistent with expectations derived from previous atomic force microscopy measurements showing a reduction in Young’s modulus of CHO-K1 cells after nsPEF exposures [44]. Further discussion of the link between cytoskeletal disruption and reduction of Brillouin frequency shift is presented later in this report.

### Supporting fluorescence microscopy imaging and cytoskeletal disruption

Independent confirmation of the biophysical mechanisms inducing cellular mechanical changes was pursued using fluorescence microscopy. Two commonly observed effects of nsPEF exposure are plasma membrane permeabilization, followed by cytoskeletal disruption. These two effects are widely believed to be causally linked. Initial disruption of the plasma membrane, caused by the nsPEF exposure, is immediately followed by a rapid influx of calcium into the cell as well as a release of intracellular calcium stores from the mitochondria and endoplasmic reticulum [45, 46]. Once released, calcium ions play a critical role in regulating the structures and function of cytoskeletal structures, specifically through depolymerization of both actin and microtubule filaments [47, 48]. Damage to these structures should lead to changes in the cell mechanical properties (and therefore, the Brillouin frequency shift). This process represents the most likely cause of nsPEF-induced mechanical change [49, 50]. We used fluorescence microscopy to confirm both membrane permeabilization using a dye uptake study, as well as both actin and microtubule damage using two cell lines transfected with fluorescent RFP-labeled α-actinin proteins and mEmerald labeled tubulin, respectively.

#### Visualizing membrane permeabilization through YO-PRO-1 uptake

**Fig. 3:**
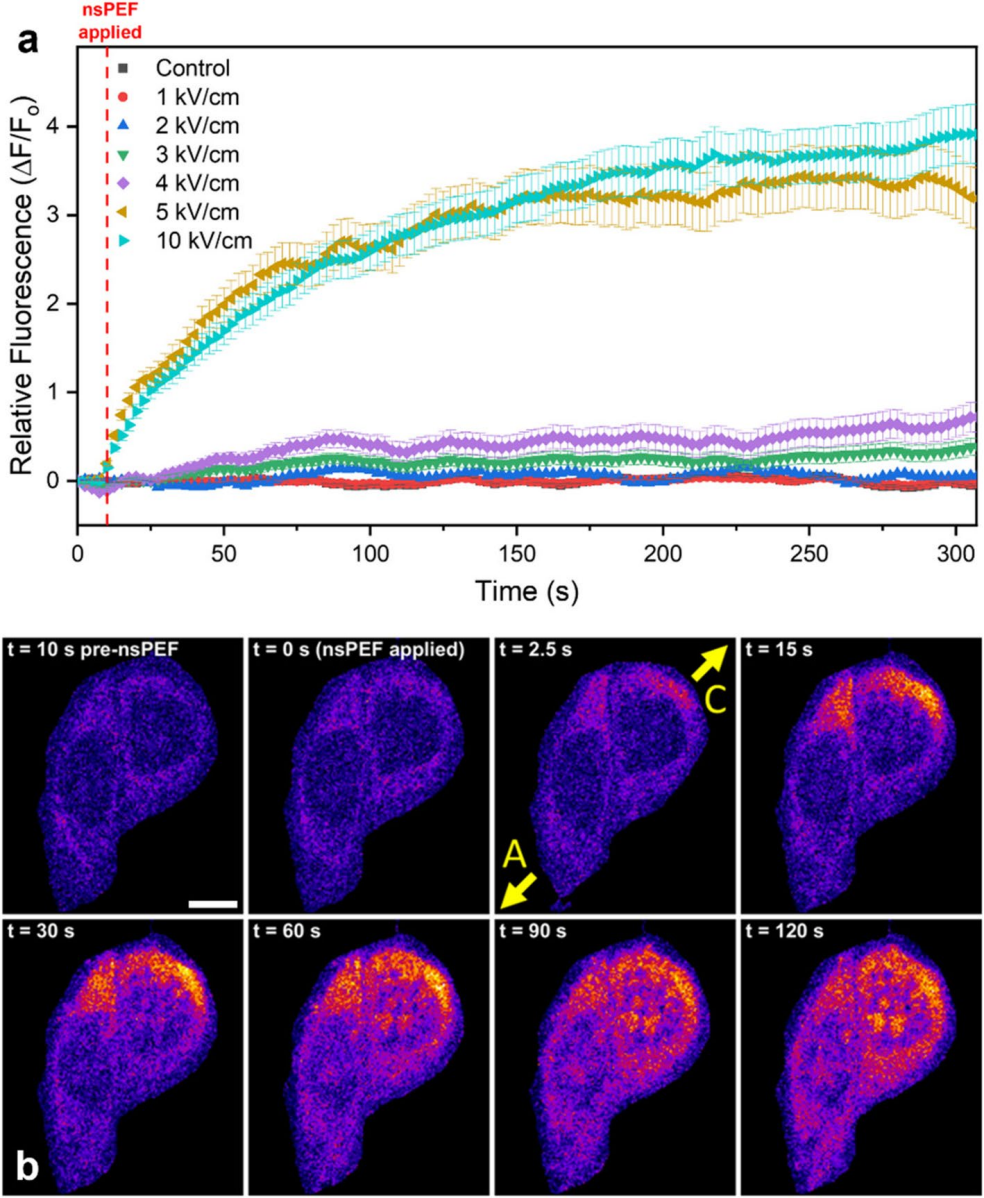
YO-PRO-1 dye uptake following nsPEF exposure. (Top) Average relative change in fluorescence (∆F/F_o_) taken from individual cells following nsPEF exposure of 1–10 kV/cm. (Bottom) Fluorescence time series example showing YO-PRO-1 dye uptake and a cathode-dependence directionality of dye influx. Yellow arrows with letters indicate the (A) anode and (C) cathode side of the electrode positions. Scale bar represents 5 μm

YO-PRO-1 is a dye that is commonly used for nsPEF-based membrane permeabilization experiments to visualize membrane disruption. This dye is generally excluded from cells with intact plasma membranes; however, the dye can permeate through a damaged membrane, and thus, increased intracellular fluorescence can be used for visualization of membrane damage [32, 51, 52]. YO-PRO-1 uptake was observed for various nsPEF intensities to verify membrane permeabilization and validate exposures against previous reports [33, 51]. YO-PRO-1 uptake is indicated by an increased fluorescence emission from within the cells, an example of which is shown in Fig. 3b, along with average fluorescence measurements for exposures up to 10 kV/cm (Fig. 3a). This data indicates that the membrane became permeabilized above a threshold pulse intensity and that dye uptake had a direct dependence on nsPEF intensity above this threshold. Asymptotic behavior of the fluorescence intensity was observed, and the minimum nsPEF threshold for observable YO-PRO-1 uptake was approximately 3 kV/cm. Both the nsPEF intensity dependence and asymptotic fluorescence curve are as expected [32, 33].

#### Visualizing nsPEF induced cytoskeletal changes

DIC and fluorescent image series were acquired over a period of five minutes following exposure to nsPEF to observe morphological and cytoskeletal changes. Fluorescence measurements were acquired from randomly selected cells following application of a single nsPEF stimulus. Cells were randomly selected with no preference for morphology or size; however, as most cells exhibited minimal swelling and only minor bleb formation [53], cells that exhibited extreme swelling and blebbing were exempted from inclusion in this study. Such cells often had large fluctuations in their Brillouin frequency shifts, likely due to cell motion and subsequent focal drift across membrane or bleb interfaces. Less than 10% of cells were excluded by these criteria. We did not attempt to characterize the volumetric changes from cells, as this has been performed extensively in the literature [32, 33, 45].

**Fig. 4:**
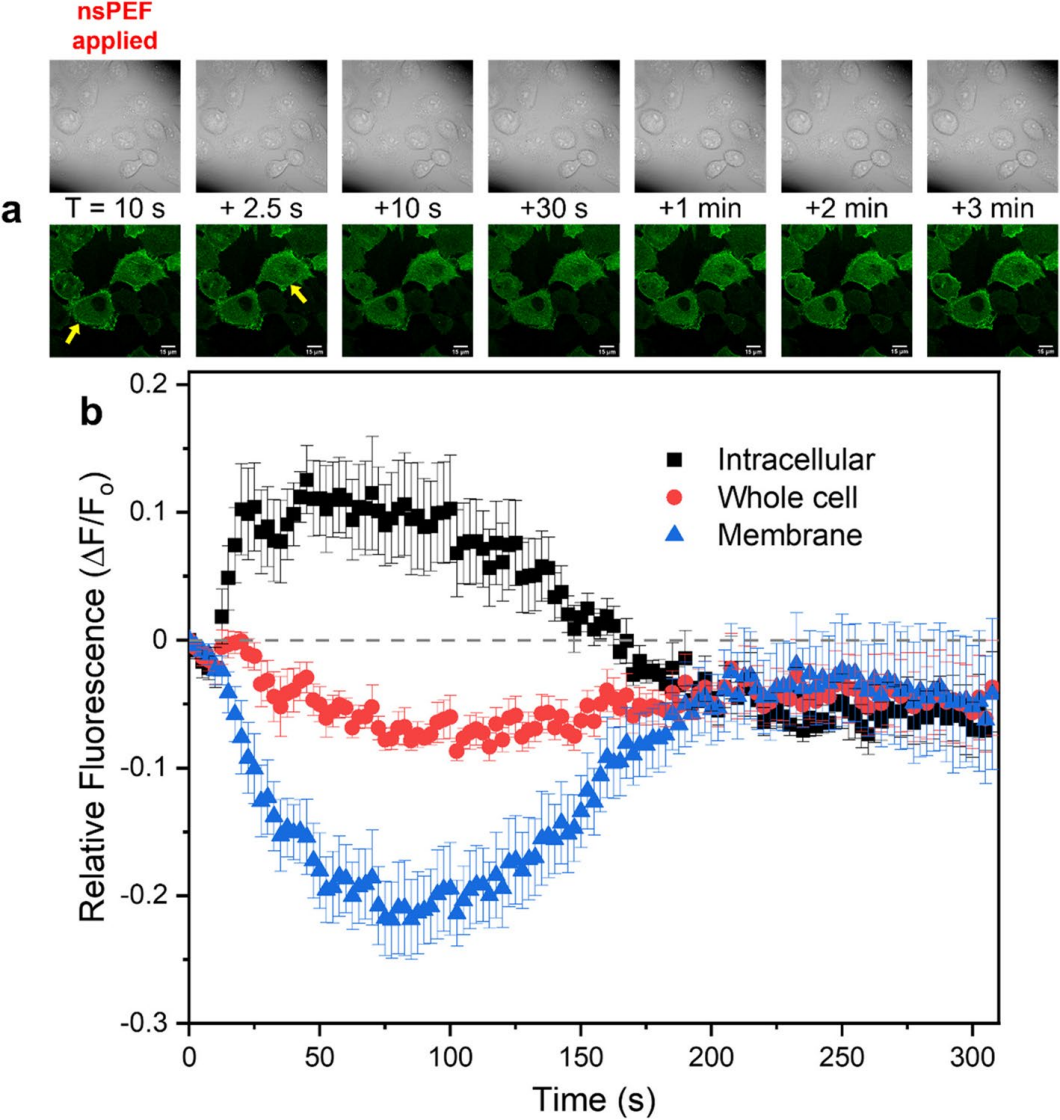
**a** DIC and fluorescence images from time series showing changes in actin distribution within cells exposed to a single 5 kV/cm nsPEF with yellow arrows pointing toward areas of membrane bound cortical actin and intracellular diffuse actin fluorescence. **b** Average change in relative fluorescence (∆F/F_o_) measurements from cells within the field of view for the above time series images. Intracellular measurements were recorded with traces inside the cell membrane, whole cell measurements were recorded from traces outside the cell membrane, and membrane specific measurements were recorded from inside of the annulus of the previous two traces

The most notable changes occurred in cells transfected with RFP-labeled α-actinin. Figure 4a provides an example of images from a single series recorded with DIC and fluorescence, respectively, from RFP-labeled α-actinin CHO-K1 cells. The first frame was acquired immediately prior to nsPEF stimulus, with further frames extending to three minutes following nsPEF stimulus. Frames from the ten seconds prior to exposure were omitted, as they provided no additional information. Analysis of images taken just above the cell basal layer showed a rapid decrease in membrane-bound cortical actin fluorescence, as well as an increase in intracellular fluorescence from what we hypothesize to be misaligned, and fragmented fibers that moved toward the center of the cells immediately following nsPEF stimulus. The membrane-bound cortical actin measurements were taken as the fluorescence intensity between two concentric traces about the membrane, with one along the inside (labeled as intracellular measurement) and one just outside (whole cell measurement) the cell membrane. Intracellular actin measurements were then recorded from within the inner trace. Image series indicate that reduction in cortical actin fluorescence starts as early as 2.5 s after stimulus application. Most cells showed sudden and substantial reduction in membrane fluorescence and noticeably higher fluorescence intensity from diffuse actin within the cell. The observed change in fluorescence intensity reaches a maximum at approximately 30 to 45 s, after which the fluorescence distribution begins to return to the initial arrangement, except for bright actin spots throughout parts of the cell. The plot provided in Fig. 4b provides an example of relative change in fluorescence (∆F/F_o_) recorded from intracellular actin and cortical actin following application of a 5 kV/cm stimulus. No apparent changes were observed in cells transfected with the mEmerald tubulin-labeled proteins. Our results indicate that rapid changes occur in the actin network of cells immediately following application of the nsPEF stimulus and provide a reasonable explanation that actin disruption is a primary driver behind mechanical changes within the cell.

## Discussion

This report demonstrates the capability of Brillouin spectroscopy for monitoring biomechanical properties of cells and subcellular components and their dynamic changes in response to fast-acting stimuli. Motivation for this study was driven from previous reports of nsPEF induced changes in the cytoskeleton [33, 51, 54], AFM-based reports of nsPEF-induced reduction of cell stiffness [44], a recent publication relating cytoskeletal structural changes to cell nuclear mechanics [40], and a recent quantitative phase imaging report suggesting a reduction in the shear modulus of cells induced by a series of applied nsPEFs [53]. We chose nsPEFs as our stimulus because they rapidly induce cytoskeletal disruption through a controlled, well-characterized process. Furthermore, PEFs have become an important scientific and clinical tool with applications in several cancer treatment therapies [55, 56], gene transfection techniques [57], and even in food treatment and processing [58] indicating that our results could be of interest across a variety of fields.

The data presented here indicate that the cytoplasm, nucleoplasm, and nucleoli exhibit not only characteristically different properties, but also different responses to the applied stimulus. For example, the longitudinal modulus of the cytoplasmic region, as indicated by Brillouin frequency shift, experienced a greater reduction than the nucleoplasm following an identical nsPEF exposure. By contrast, nucleoli exhibited much less change, even when compared to measurements from within the rest of the nuclear envelope. Furthermore, we observe that the changes in mechanical properties occur quite rapidly following the nsPEF application but require between 2 and 5 s to be measurable beyond one standard deviation from mean and are therefore not instantly observable. The changes in cell properties are, however, detectable via spectral analysis long before cell swelling can be observed via microscopic imaging, suggesting that Brillouin spectroscopy is more sensitive to micromechanical changes than perturbative or strictly visual methods. These results are supported by our fluorescence imaging, which indicates a drastic reduction in cortical actin along the membrane following nsPEF exposure, as well as an increase in intracellular fluorescence, likely from disordered and fragmented actin within the cytoplasmic region of the cell. This outcome is consistent with our expectations, as nsPEF stimulus has been shown to disrupt the cell membrane, which leads to further downstream disruption of other cytoskeletal structures in the actin cytoskeleton and microtubule network. These results agree with previous reports indicating that depolymerization of the actin network should lead to greater changes in the cytoplasmic region of cells as compared to the nucleus and nucleoli, where actin is not present [36], and that disruption of the actin cortex should lead to reduced stiffness from within the nucleus of cells as the connections between nucleus and membrane are reduced [40] along with the softening of the actin cortex. Furthermore, compartment-specific measurements of the Brillouin frequency shift suggests that different intracellular regions exhibit different susceptibilities to this stimulus, which may be related to their material and structural properties. Alternatively, this difference in Brillouin frequency shift magnitude could simply be a result of changes in the various cytoskeletal connections, as measurements from the cytoplasmic region would be more greatly impacted by actin disruptions than within the nucleus, due to disruption of actomyosin contractility and loss of local filament connections [40]. Furthermore, the nucloli of the cells may also swell slightly or experience a slight increase in volume as the actin filaments are disrupted, contributing to or even causing the more muted decrease in Brillouin frequency shift that was observed there. Recent investigations into the resolution limits of Brillouin spectroscopy have brought to light that measurements at this scale should likely be considered more from a local averaged spatial unit rather than a precise sub-micrometric measurement [59]. This local averaging is due to combination of the large collection angle of high NA microscope objectives as well as inhomogeneities within the sample, particularly when local acoustic phonons have much longer mean free path than the probed volume that convolve to generate a broadened Brillouin spectral signal [29, 37, 60, 61].

Fluorescence imaging was used to examine changes in the actin and microtubule structures within cells subject to these exposures. Actin and microtubules are two of the primary components of the cell cytoskeleton; disruption of one or both structures should cause immediate changes in cell micromechanics. Time series images showed drastic changes and redistribution of actin, alongside minimal differences in microtubule structures. This data indicates that, within the time frame of our Brillouin spectroscopy investigations (≤ 90 s following nsPEF exposure), the observed reduction in Brillouin frequency shift magnitude, and thus longitudinal modulus, is primarily driven by cytoskeletal rearrangement tied to disruption of actin structures and not disruption of microtubules. We note that actin stress fibers could be visually inspected along the basal layer of the cell at the glass substrate interface. The basal layer showed a reduction in fluorescence intensity following the same trend as when focused further above, however, the stress fibers were seen to remain intact and mostly unchanged. Regarding the dye uptake study, we note that there is little to discern between 5 kV/cm and 10 kV/cm within the first two minutes following exposure. This would imply that the intracellular effects would likely be similar between these two exposure conditions, yet the Brillouin measurements indicate very little change at 10 kV/cm which is in agreement with previous studies regarding cellular swelling and blebbing following single 600 ns nsPEF exposures [62]. Additional studies may be necessary to determine the sensitivity threshold of Brillouin spectroscopy to specific intracellular disturbances and cytoskeletal rearrangements either with lower pulse intensities or with targeted or natural disruption methods that do not toxify the cellular environment, such as the natural cytoskeletal rearrangement that occurs during the cell life cycle and reproduction.

We note that we did not detect morphological changes, nor did we detect discrepancies in the Brillouin spectra collected from cells during control investigations; however, the selected wavelength of 532 nm has great potential to cause harm to cells and is not appropriate for longer-term cellular investigations. Alternative probe wavelength should be considered when developing longer-term Brillouin investigations into biological materials. Wavelengths that have less absorption in biological samples and are less likely to cause damage, such as 660 or 780 nm are recommended to ensure that the measurement technique itself does not impact the sample [43, 63].

This report focuses on short-term, near-immediate changes in mechanical properties of cells and subcellular structures induced by a transient electrical stimulus, while other reports have focused on changes over much longer time periods (≥ 15 min) targeting specific parts of cells using chemical agents that cannot be easily removed from the environment. We selected nsPEF exposures, in part, because nsPEF can be considered a “messy” stimulus that can induce myriad changes in cell structures and function. Therefore, additional studies are warranted for monitoring cytoskeletal and mechanical property changes over longer periods following nsPEF exposures to compare and assess against those from specifically-targeted changes such as those presented by Alisafaei, et al. and Zhang, et al. [40, 64]. Of particular interest to future Brillouin-related biological studies is that many changes in cellular mechanics are associated with changes in osmolarity or water content within cells. We have worked to combine Brillouin spectroscopy with other powerful optical investigation tools to further explore this topic. Preliminary results from that study (not shown here) indicate that intracellular water content in CHO-K1 cells is likely responsible for approximately 50% of the measured reduction in Brillouin frequency shifts following nsPEF exposures with the other half attributed to cytoskeletal damage and structural change [65].

## Conclusion

In conclusion, we have applied recent advances in Brillion spectroscopy to achieve rapid assessment of subcellular biomechanical changes on a microscopic scale. Our results demonstrate the unique capabilities of Brillouin microscopy and suggest the need for further adoption of the technology to study rapidly evolving cytomechanical structures in real time. Coupled with Raman spectroscopy, the reported system can be used to simultaneously observe fast mechanical and chemical changes within intracellular compartments. We expect Brillouin micro-spectroscopy to vastly expand the scope of biomechanical research.

## Materials and methods

### Cell culture and sample preparation

CHO-K1 cells (ATCC® CCL-61™, Chinese hamster ovary) were cultured and maintained following the recommended protocol. Cultures were maintained at 37 °C with 5% CO_2_ in air with 95% relative humidity, propagated in Kaighn’s Modification of Ham’s F-12 Medium (F-12 K Medium, ATCC® 30-2020™) supplemented with 10% fetal bovine serum (FBS, ATCC® 30-2020™), 2 mM L-glutamine, and 1% volume 100 U/mL penicillin/streptomycin (ATCC® 30-2300). Samples were prepared by seeding 20,000–30,000 cells in a culture dish with a glass bottom coverslip (P35GC-1.5-14-C, Mattek Corporation, Ashland, MA). The following day, depleted culture media was replaced with fresh media, and dishes incubated for an additional 30–60 min. Imaging was performed in cell culture media as opposed to standard imaging buffer solution, as the proteins in cell media can help to inhibit cell swelling. This ensures that changes in the Brillouin shift occur due to disruption of the cytoskeleton, rather than cell swelling.

### Brillouin-Raman microspectroscopy system

**Fig. 5:**
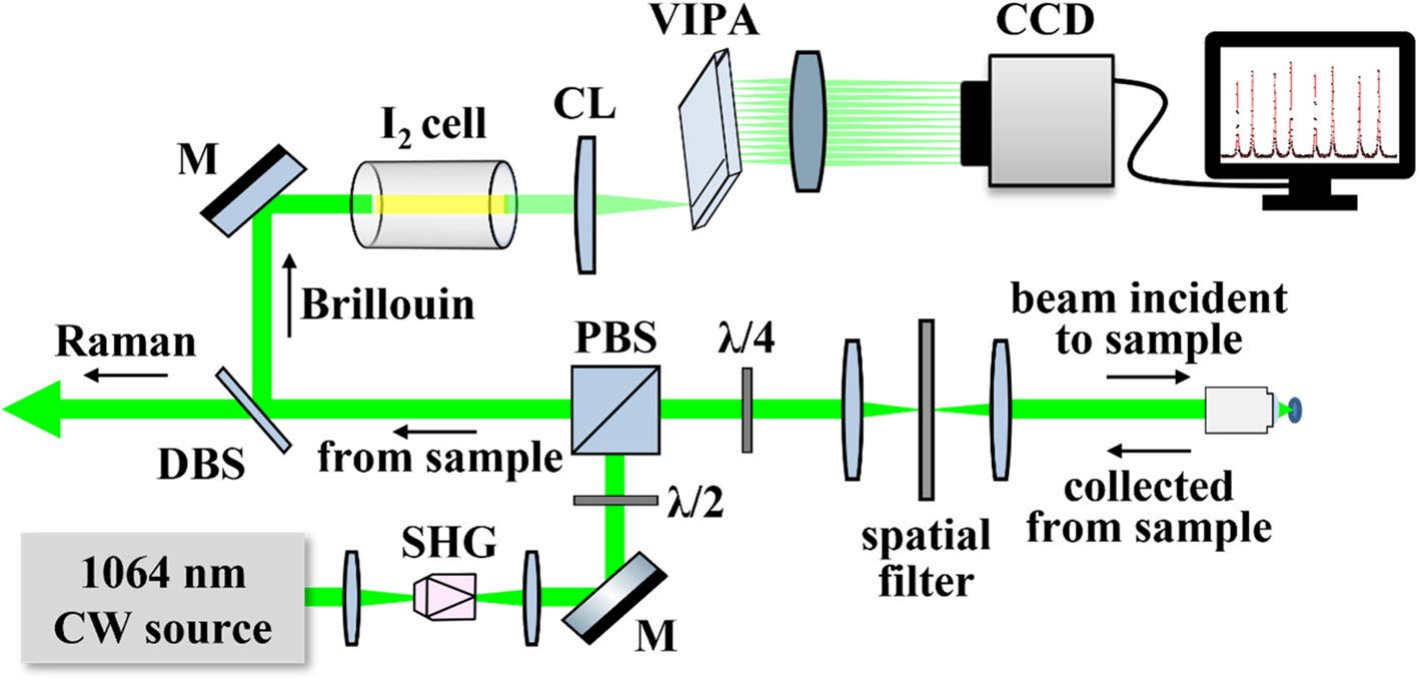
Schematic diagram of VIPA-based dual micro-Brillouin-Raman spectroscopy system. Abbreviations and labels: SHG second harmonic generation of 532 nm beam, λ/2 half wave plate, M mirror, PBS polarizing beam splitter, DBS Raman dichroic beam splitter, I_2_ Iodine molecular absorption cell, CL cylindrical lens, and CCD spectral imaging camera

A custom Raman-Brillouin microscopy system was built based on previous reports [66–69]; however, the laser source was replaced with an ultra-stable custom-build laser system to minimize the source of noise in Brillouin spectroscopy measurements. A schematic of the system is provided in Fig. 5. In brief, the microscope system was built around a tunable, ultra-narrow (< 1 kHz) single-frequency laser (Koheras ADJUSTIK/BOOSTIK Y10; NKT Photonics) with 1064 nm center wavelength and 2.1 W maximum output power. A magnesium doped PPLN crystal (MSGH1064-1.0-20; Covesion, Ltd.) was used for second harmonic generation (SHG) to produce an output wavelength of 532 nm with up to 150 mW maximum output power. The laser was coupled into the rear port of an inverted microscope (IX73; Olympus Corp.) and focused to the sample using a 1.42 NA 60X objective lens (UPLXAPO 60XO; Olympus Corp.). The spatial resolution of the mechanical imaging system is primarily limited by the mean free path of vibrational wavelengths and the numerical aperture of the objective lens. In our confocal setup, the lateral and axial resolution of the Brillouin acquisitions were estimated to be approximately 500 nm × 500 nm × ~1 μm, respectively, based on the finite numerical aperture diffraction limit, local vibrational length scales [29, 61], and spectral measurements across reflective surface interfaces [37]. The axial resolution for Raman measurements was expected to be approximately 25% larger, as the confocal pinhole for Raman photons was selected to be slightly larger than the diffraction limited Airy disk pattern for increased signal strength. Light was collected from the sample in a 180° backscattered (epi-illumination type) configuration by the same objective and separated into respective Raman and Brillouin analysis paths by a dichroic beam splitter (RazorEdge LPD02-532RU; Semrock). Appropriate pinholes were placed in each path to provide confocal gating of the scattered photons. Raman signal was passed through a narrow band notch filter, and directed into a spectrometer (Shamrock 303i, Andor Technology Ltd.), where it was dispersed by a 600 line/mm reflective grating and collected by a CCD camera (iDus 420, Andor Technology Ltd., cooled at − 70 °C). Brillouin signal was passed through an I_2_ absorption cell (GC19100-I; ThorLabs, Inc.) to a virtually imaged phase array (VIPA)-based Brillouin spectrometer and collected by an EMCCD camera (Ixon Ultra 888; Andor Technologies Ltd., cooled at − 100 °C). This arrangement uses an I_2_ gas cell as an ultra-narrow absorption-based notch filter, removing background Rayleigh scattered light that would otherwise saturate the detector and cover the much weaker Brillouin peaks [70]. The VIPA used in our spectrometer had a free spectral range (FSR) of 29.95 GHz (OP-6721-3371-2; Light Machinery, Inc.). Our Brillouin spectrometer exhibited a spectral contrast of -80 dB with spectral resolution of $\delta \nu$ = 485 ± 12 MHz, as measured by the spectral width of the laser projected through the VIPA. The repeatability of Brillouin shifts, as measured from individual target components, was generally ≤ 10 MHz, determined by the standard deviation of repeated measurements from within the same cell over two minutes, providing a relative precision of ~ 0.14%.

### Nanosecond electric pulse (nsPEF) system and cell stimulus

The external stimulus applied to the cells was a 600 ns duration electrical impulse driven by a high-power pulse generator and delivered via a custom microelectrode arrangement [51, 71]. This stimulus was chosen because it is known to rapidly depolymerize the cytoskeleton, allowing demonstration of real-time cytomechanics using Brillouin imaging [32, 33, 44]. A pair of tungsten electrodes, 125 μm in diameter spaced approximately 200 μm apart, were placed on opposite sides of target cells approximately 50 μm above the petri dish cover slip at a 45° angle using a micromanipulator (MPC-200; Sutter Instruments). Experiment timing was controlled using a digital delay pulse generator (DG535; Stanford Research Systems, Inc.) through which a single-shot trigger button was used to initiate both Raman and Brillouin spectral measurements. After a preset delay of 10 s, a single nsPEF pulse was delivered. Pulse delivery was monitored and recorded (pulse amplitude and width) using a 100x voltage probe (P5100A; Tektronix) coupled to an oscilloscope (TDS3054C; Tektronix).

### Fluorescence microscopy

#### YO-PRO-1 dye uptake

All dye uptake studies were performed over a total of five minutes and 10 s. The first 10 s of exposures were taken prior to application of nsPEF to allow for a baseline and background fluorescence measurement in each image series. The following five minutes of image acquisition were acquired to provide a relatively long-term study of dye uptake to determine membrane recovery. Membrane permeabilization and dye uptake experiments were conducted over a range of nsPEF intensities from 0 kV/cm to 20 kV/cm.

#### Cytoskeletal imaging

Changes in two of the major cytoskeletal elements, actin, and microtubules, were visually assessed using separate CHO-K1 cultures. These cultures were separately transfected with mEmerald-labeled tubulin and RFP α-actinin for the observation of the microtubule filaments and actin network structures. Additionally, z-stack image series were acquired pre- and post-nsPEF application to check for morphological changes or redistribution of the fluorophore-labelled structures. Images were post-processed using ImageJ FIJI software (ImageJ 1.53c, National Institutes of Health, USA). Cells were randomly selected, and their borders manually traced for fluorescence data recording and analysis. Cell mean fluorescence and time series relative change in fluorescence (∆F/F_o_) were determined and assessed after background subtraction for each cell measured across every timepoint. Background measurements were taken from an average of 3 to 5 randomly selected regions of each image series where cells were not present. All fluorescence intensity values are given in terms of change in relative fluorescence, with respect to the initial fluorescence measurement of each cell.

### Signal acquisition and analysis

Single cells were located via brightfield microscopy and positioned such that the target region was centered about the focal volume of the laser. Specific cell components were visually located using differential interference contrast (DIC) imaging. The axial focus of the Brillouin probe should theoretically match that of the imaging plane for the microscope objective. To verify that this was true, we slowly adjusted the focus of the microscope to optimize the image of a resolution target at the imaging camera and ensured that the reflection of the Brillouin probe from the target came to a tight focus at both the Brillouin spectrometer camera and the microscope imaging camera. The cell position, with respect to the laser, could then be determined by opening the rear-port shutter of the microscope and visually comparing the cell location to the laser spot reflected by the glass substrate and compared against a digital crosshair available in the camera software (Pylon 6.2.0 Camera Software Suite; Basler AG, Ahrensburg, Germany). Once a target location was selected, the DIC analyzer and Nomarski prism were removed from the optical path so signal acquisition could begin. The laser spot location was verified periodically between experiments; no observable positional variance was ever detected. However, cells are dynamic in nature and thus may move during data acquisition. Therefore, brightfield images were taken of each cell both before and after exposures. Images were then compared after data acquisition to check that no significant movement had occurred during the acquisition and that the laser voxel was still within the target region as reported by the software-based crosshair. Data series were excluded from analysis if the images indicated that cell movement, swelling, blebbing, or contraction caused the internal target compartment to move more than ~ 1.5 μm between the initial and final image. In addition to monitoring sample motion using brightfield images, the Raman spectra were useful for indicating axial focal drift and cell motion, particularly when cells moved away from the laser focus. Raman spectra acquired from outside of a cell contained a large background fluorescent signal from the growth medium, providing a useful indicator to further ensure proper placement of the focal zone.

Raman and Brillouin spectra were acquired from randomly selected cells across several days, with individual cells selected per exposure and per target region, such that no two-measurement series were recorded from the same cell. Spectra were acquired for a period of 100 s. nsPEF was applied at t = 10 s such that all trials had an initial 10 s baseline measurement. Our time resolution for Brillouin and Raman measurements were ∆t = 0.5 and ∆t = 5 s, respectively. Signal acquisition continued for 90 s post-exposure to allow for observation of any changes in the mechanical properties of the target region immediately following the stimulus. All Brillouin measurements were normalized to the average of their own 10 s baseline measurements for comparison. As all measurements indicate very small differences, generally < 2% ${\nu }_{B}$, all time-series measurements are presented here as percent change, or percent value from the 10 s baseline average.

Laser power at the sample was kept below 4 mW to avoid localized heating and cell phototoxicity. It has previously been reported that cell damage may occur when an average of ~ 3 J energy has been delivered to cells using 532 nm light [63]. At our power output of < 4 mW this energy delivery would occur after roughly 13 min of continuous exposure, or close to 8 times longer than our 100 s Brillouin study. For verification, we conducted a preliminary test series that showed no morphological changes or cell blebbing were present near the focal volume in cells after a continuous 5-minute exposure at this power level. Furthermore, control measurements indicated no immediate changes in Brillouin spectral measurements. Spectral acquisition times were 0.5 s for Brillouin spectra and 5 s for Raman spectra. Each trial consisted of a 100 s laser exposure, for a total of 220 spectral measurements (200 Brillouin and 20 Raman), with the nsPEF exposure coinciding with the beginning of the 21st and 5th measurement for Brillouin and Raman spectra acquisitions, respectively.

Spectra were plotted using OriginPro 2020b (OriginLab, Northampton, MA.). Brillouin spectral analysis was performed using a custom Python script (Python 3.8.5). Through this analysis, the Brillouin peaks were individually located and fit Lorentzian function using a least-squares fitting protocol. The overall Brillouin frequency shift was then determined as half the distance between the respective Stokes and anti-Stokes peak centers. Raman spectra from before exposure and 90 s after were compared to check for any differences in peak intensities and relative peak height ratios following background subtraction. Background subtraction was completed using a custom MATLAB code based on the modified polynomial fit method presented by Lieber and Mahadevan-Jansen [72].

## Data Availability

The data that support the findings of this study are available from the corresponding author upon reasonable request.
